# Renal and Cardiovascular Metabolic Impact Caused by Ketogenesis of the SGLT2 Inhibitors

**DOI:** 10.3390/ijms24044144

**Published:** 2023-02-18

**Authors:** Ariana P. Vargas-Delgado, Estefania Arteaga Herrera, Cesar Tumbaco Mite, Patricia Delgado Cedeno, Maria Cristina Van Loon, Juan J. Badimon

**Affiliations:** 1 AtheroThrombosis Research Unit, Mount Sinai Heart, Icahn School of Medicine at Mount Sinai, One Gustave L. Levy Place, New York, NY 10029, USA; 2 Instituto Ecuatoriano del Corazón (IECOR), Guayaquil 090513, Ecuador

**Keywords:** sodium–glucose cotransporter type 2 inhibitors, type 2 diabetes mellitus, heart failure, ketone bodies, free fatty acids, chronic kidney disease, arterial stiffness

## Abstract

Sodium–glucose cotransporter type 2 inhibitors (SGLT2i) are glycosuric drugs that were originally developed for the treatment of type 2 diabetes mellitus (T2DM). There is a hypothesis that SGLT2i are drugs that are capable of increasing ketone bodies and free fatty acids. The idea is that they could serve as the necessary fuel, instead of glucose, for the purposes of cardiac muscle requirements and could explain antihypertensive effects, which are independent of renal function. The adult heart, under normal conditions, consumes around 60% to 90% of the cardiac energy that is derived from the oxidation of free fatty acids. In addition, a small proportion also comes from other available substrates. In order to meet energy demands with respect to achieving adequate cardiac function, the heart is known to possess metabolic flexibility. This allows it to switch between different available substrates in order to obtain the energy molecule adenosine triphosphate (ATP), thereby rendering it highly adaptive. It must be noted that oxidative phosphorylation in aerobic organisms is the main source of ATP, which is a result of reduced cofactors. These cofactors include nicotine adenine dinucleotide (NADH) and flavin adenine dinucleotide (FADH2), which are the result of electron transfer and are used as the enzymatic cofactors that are involved in the respiratory chain. When there is an excessive increase in energy nutrients—such as glucose and fatty acids—which occur in the absence of a parallel increase in demand, a state of nutrient surplus (which is better known as an excess in supply) is created. The use of SGLT2i at the renal level has also been shown to generate beneficial metabolic alterations, which are obtained by reducing the glucotoxicity that is induced by glycosuria. Together with the reduction in perivisceral fat in various organs, such alterations also lead to the use of free fatty acids in the initial stages of the affected heart. Subsequently, this results in an increase in production with respect to ketoacids, which are a more available energy fuel at the cellular level. In addition, even though their mechanism is not fully understood, their vast benefits render them of incredible importance for the purposes of further research.

## 1. Introduction

Sodium–glucose cotransporter type 2 inhibitors (SGLT2i) are glycosuric drugs that were originally developed for the treatment of type 2 diabetes mellitus (T2DM) [1]. However, these drugs have progressed and developed in numerous ways from their initial purpose. These drugs have been shown to possess marked cardiovascular, renal, and metabolic benefits in both diabetic and non-diabetic populations, among other such gains. From the publication of the EMPA-REG OUTCOME and DECLARE-TIMI 58 studies in 2015 and 2019, respectively, their effectiveness was demonstrated among the population with T2DM [1,2]. Further, the EMPATROPISM trial evidenced their benefits among the non-diabetic population; in addition, there have been new publications that also corroborate such advantages. This has thus generated a new trend in the various treatment guidelines, which are utilized by the main scientific societies in the world [1,2]. Therefore, it appears relevant to us to objectify the mechanisms and effects of the ketogenesis that are caused by this novel group of drugs that improve myocardial efficiency [1].

The heart possesses a “metabolic flexibility” that allows it to change its substrate in order to meet its energy demands, as well as to achieve adequate cardiac function. During ischemia, the heart stops receiving oxidizable substrates and oxygen, which implies that glycolysis occurs at the expense of the glycogen present in cardiomyocytes. Therefore, this implies that there is a reduction in lactate production and thus a favoring of intracellular acidosis [3,4].

Additionally, it has been hypothesized that SGLT2i are drugs capable of increasing the ketone bodies and free fatty acids that will serve as the necessary fuel, instead of glucose, for cardiac muscle requirements [3].

It is also believed that the ketogenic properties of SGLT2i could explain their antihypertensive effects, independent of renal function. Therefore, it is likely that natriuresis and the reduced volume load explain, in part, the protective effects of SGLT2i with respect to heart failure (HF) [5].

## 2. Function of Glucose Transporters and Sodium–Glucose Cotransporters at the Renal Level

Sodium–glucose cotransporters are a family of transmembrane proteins located in different parts of the body, whose main function is the reabsorption of sodium and glucose (which is where much focus has been directed in recent years). Under normal conditions, approximately 180 g of glucose are filtered and reabsorbed by the kidney. In order to mobilize glucose at the intracellular level against the gradient, the presence of sodium-glucose cotransporters type 1 and 2 (specifically SGLT1 and SGLT2, respectively), is necessary [6].

It is important to know that SGLT1 receptors are found predominantly in the small intestine and, to a lesser extent, in the kidneys; moreover, they are found in segment S3 of the convoluted tubule. They are considered transporters of high affinity for the reabsorption of glucose when filtered by the glomerulus, but they are also of low capacity due to the fact that they only reabsorb approximately 10% of the glucose. Unlike the SGLT2 receptors that are primarily located in the kidney at the level of the S1 segment of the apical membrane of the proximal convoluted tubule, instead they are low-affinity transporters with a high capacity for glucose reabsorption, which is due to the reabsorption of approximately 80% to 90% of the glucose that is filtered by the glomerulus [7].

**Figure 1:**
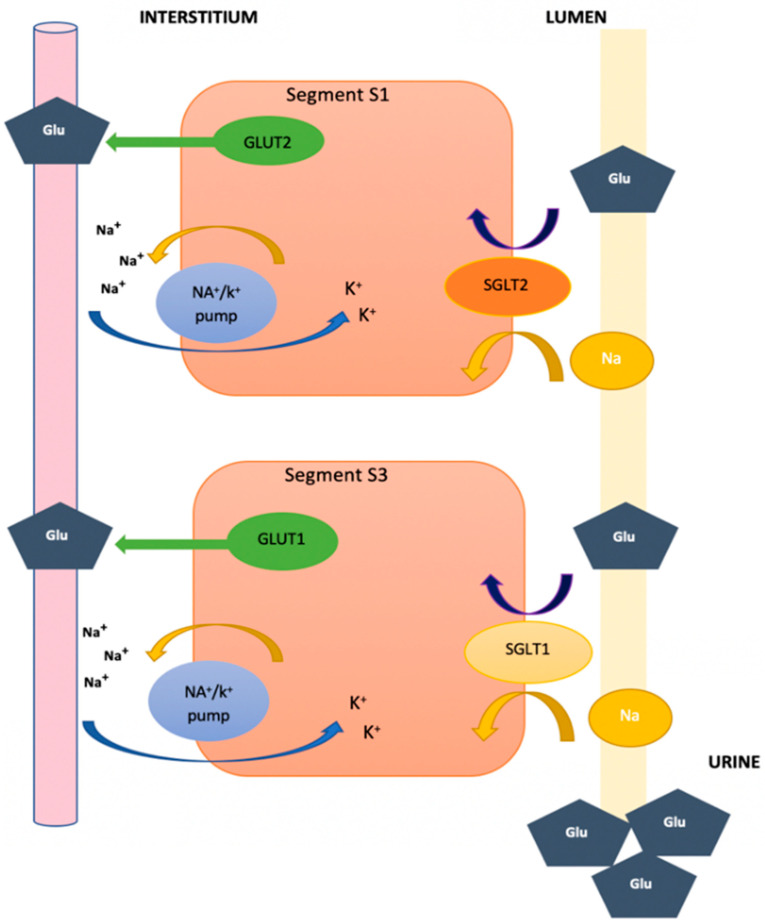
Glucose reabsorption in the proximal convoluted tubule. Edited from ref. [8].

Reabsorbed glucose is then released into the circulatory system through facilitated diffusion thanks to glucose transporters type 1 and 2 (specifically GLUT1 and GLUT2, respectively), as illustrated in Figure 1, which are located in the epithelial cells, but in the direction of the basolateral membrane [5]. When the amount of serum glucose exceeds 180–200 mg/dL (11.11 mmol/L)—thus surpassing the reabsorption capacity of the kidney—glycosuria occurs as a result, which is also an occurrence that is observed in patients with uncontrolled T2DM [3,4,7].

## 3. Energy Metabolism in the Heart

Under normal conditions, the adult heart consumes around 60% to 90% of the cardiac energy that is derived from the oxidation of free fatty acids. In addition, a small proportion of this energy is also derived from other available substrates. In order to meet its energy demands, the heart is known to have metabolic flexibility to achieve adequate cardiac function. This allows it to switch between different available substrates in order to obtain the energy molecule adenosine triphosphate (ATP), thus rendering it highly adaptive [3].

On account of glucose being extremely important with respect to these processes, specifically due to it being one of the main substrates catabolized by the heart, glucose transporters that will help the heart maintain its metabolic functions properly, as well as remain in balance, are important. This is unlike in other certain situations such as hypoxia, where the heart’s metabolism depends on glucose in the case of an emergency situation. Indeed, this substrate is regulated by sodium–glucose cotransporters in addition to the glucose transporters GLUT1 and GLUT4. These specific transporters have high affinity for glucose and are present in tissues regulated by insulin action, such as cardiac muscle, skeletal muscle, and adipocytes [3,4].

In pathological processes—such as HF, diabetic cardiomyopathy, and ventricular hypertrophy—there is an alteration at the level of glucose transport. This results in the production of excessive availability with respect to glucose transport, which thus generates an exaggerated contribution of energy that finalizes in saturating the cellular environment. As a result of this overload, the heart will seek to resort to other metabolic cycles. Moreover, SGLTs at the cardiac level are most likely not related to energy production, but they are part of the intracellular processes that produce reactive oxygen species (ROS), which, under pathological conditions, can trigger and produce greater oxidative stress [4,9].

During embryonic development, the fetal heart utilizes glucose as its primary reserve energy substrate. This mechanism subsequently changes at birth, which, under normal conditions, utilizes free fatty acids (FFA) as its main source for energy; furthermore, this is in addition to glucose, ketone bodies (KB), and amino acids. At the cellular level, cardiomyocytes contain cellular organelles, such as mitochondria, which are responsible for generating 95% of the energy through the synthesis of ATP: the main fuel for biochemical reactions. Furthermore, to a lesser extent, the heart also obtains energy through phosphocreatine (PCr), which represents the reserve energy [10,11].

## 4. Change in Cardiac Metabolism during Hypoxia

Cardiac activity is carried out through an energy contribution that is derived from the catabolism of fatty acids via aerobic metabolism, as well as—to a lesser extent—from anaerobic glycolysis, which is how lactate is produced from glucose. It should be noted that at the level of the mitochondrial matrix, the citric acid cycle, better known as the Krebs cycle (aerobic route), is the mechanism by which the greatest amount of free energy is released during the oxidation of different metabolites. This aerobic route consists of the catabolism of acetyl Co-A from fatty acids, which are mainly oleic and palmitic, as well as carbohydrates [12,13,14].

Glucose is broken down into two pyruvate molecules, which can take two routes: the first forms two lactate molecules under anaerobic conditions, while the second route forms acetyl Co-A under aerobic conditions. There are three main components of this aerobic metabolic pathway: the first one involves the use of substrates that are present at the cellular level, which exist through the processes of b-oxidation and glycolysis, such as free fatty acids and glucose; second is the process of oxidative phosphorylation, which occurs at the level of the mitochondrial respiratory chain, where the conversion of ADP to ATP is catalyzed and where the direct energy in cardiac processes is provided; third is the transfer and use of ATP by cardiac myofibrils [12,13].

**Figure 2:**
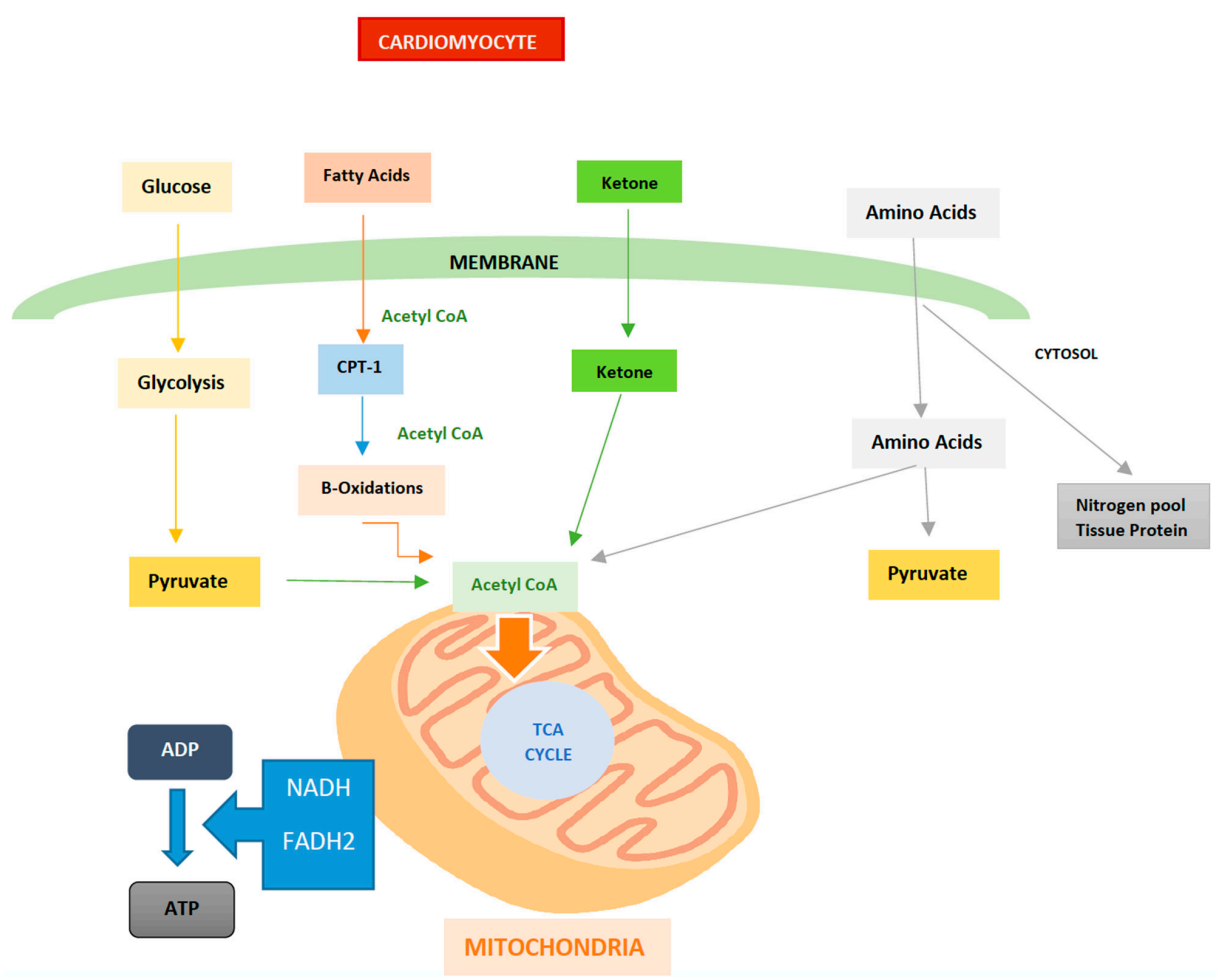
Regulation of free fatty acid oxidation and glycolysis. Author’s own work. Nicotine adenine dinucleotide (NADH) and flavin adenine dinucleotide (FADH^2^) promote oxidative phosphorylation in aerobic organisms. These are the result of electron transfer and are used as enzymatic cofactors that are involved in the respiratory chain as the main source of ATP. Glycolysis, lipolysis, and ketogenesis pathways in the mitochondria.

Oxidative phosphorylation in aerobic organisms is the main source of ATP as a result of reduced cofactors, such as nicotine adenine dinucleotide (NADH) and flavin adenine dinucleotide (FADH^2^). These are the result of electron transfer and are used as enzymatic cofactors that are involved in the respiratory chain, as seen in Figure 2 [13,14,15].

In situations where the demands increase, there is an important mechanism that occurs in order to function as a source for an energy reserve. This is achieved through mitochondrial creatine kinase, which is a molecule composed of amino acids, that is synthesized in the liver, kidneys, and pancreas. Moreover, when in its phosphocreatine form, it catalyzes the reformation of ATP [13].

In order to comply with the necessary biochemical reactions of the cell, the myocardium utilizes large amounts of energy in order to meet its constant needs. Furthermore, in order to be able to maintain this, it is important that the energy substrates it uses are available. Therefore, cardiac metabolism is capable of switching from one energy source to another in order to function properly [15].

## 5. Alternative Fuels used by the Heart

As previously mentioned, the heart depends on between 60% and 90% of the cardiac energy derived from the oxidation of fatty acids, while the remaining is at the expense of glucose and lactate. Indeed, the transport of glucose and its incorporation into cardiomyocytes are mediated by the transporter proteins GLUT 1 and GLUT 4. There are several factors that promote glucose metabolism at the cardiac level, such as hypoxia, insulin concentrations, exercise, and the availability of different substrates. The oxidation of glucose is carried out through glycolysis, which is a strictly aerobic process. In this process, pyruvate is generated in order to be oxidized and thus obtain carbon dioxide. This is performed in order to gain two ATP molecules. Under conditions in which there is insufficient oxygen, lactate is generated as a final product. This can be observed, for example, when skeletal muscle is subjected to intense effort [16].

The hypoxic heart decreases oxygen consumption in addition to the concentration of adenosine and potassium at the mitochondrial level. Prolyl hydroxylase (PHD) activity is attenuated by Krebs cycle metabolites: Succinate, isocitrate, fumarate, oxaloacetate, and pyruvate; which favors hypoxia-inducible factors α (HIF-α) subunits accumulation [17,18,19]. These changes promote a decrease in phosphorylation processes, which in conjunction with HIF-1 will reduce the activity of the Krebs cycle by inducing pyruvate dehydrogenase kinase 1 (PDK1) [20,21,22]. This enzyme inhibits the activity of pyruvate dehydrogenase to lower acetyl CoA levels, which during normal conditions is processed to citrate and acts as a fuel to the Krebs cycle [22,23].

## 6. Energy Metabolism and Oxidative Stress in T2DM and HF

An inadequate lipid deposit in different organs, such as the heart, can lead to cell damage due to the impaired capacity of cardiomyocytes with respect to storing large amounts of lipids, thereby generating lipotoxicity. This occurs due to the fact that there is an increase in the systemic level of glucose and free fatty acids. In the diabetic heart, insulin resistance activates inflammatory pathways (i.e., protein kinase C and nuclear factor K) that will limit the cardiac glucose supply, consequently leading to fatty acid oxidation. When there is a balance at the level of the substrates, it is possible to maintain the energy demand of ATP, such that the amount of waste is diminished, which is then released in the form of heat [12,24].

When there is an excessive increase in energy nutrients, such as glucose and fatty acids, and in the absence of a parallel increase in energy demand, a state of nutrient surplus—better known as an excess in supply—is created. This leads to a similar action on the part of the tricarboxylic acid cycle in order to provide energy products, such as electron donors for the respiratory chain. On the other hand, many nutrients are wasted and removed as heat, which is mediated by mitochondrial proton leakage. Therefore, an inefficient environment is created, thus favoring the excessive production of ROS and insufficient endogenous antioxidants, thereby generating oxidative stress. In addition, this process is known as a pathophysiological pathway that promotes the development and progression of HF. Furthermore, this is on account of the intracellular alteration of the ROS regulation mechanism via endogenous antioxidants, which are also severely diminished [3,24].

DM-2 and HF are one of several diseases that cause oxidative stress, which is a process of cellular deterioration that depends on the synthesis of radical oxygen species (ROS). Molecules with a high reactive capacity that damage the environment of molecules are produced by a decrease in antioxidant defenses or by an increase in oxidant molecules. Under normal conditions, there are compound–antioxidant processes that are mechanisms capable of counteracting the effects of free radicals. However, in situations such as ischemia, mechanical stretch, α-adrenergic agonists, exposure to inflammatory cytokines, angiotensin II, endothelin-1, lipotoxicity, and hyperglycemia, among others, represent predisposing factors to the formation of O_2_ radicals. This means an increase in auto-oxidative pathways, the respiratory chain, advanced glycation products, and sorbitol [16].

O_2_ radicals stimulate protein kinase C (PKC) activity, a stress signaling pathway that promotes nitric oxide synthesis and increases stress cytokines. Thus, increasing the activity of the NADPH and xanthine oxidase system. Increased activity of these enzymes leads to further increases in peroxynitric and O_2_ radicals. The overproduction of reactive oxygen species, combined with reduced antioxidant defenses, generates a pro-oxidant state that conditions oxidative damage to proteins, nucleic acids, carbohydrates, and lipids. Contributing to the development of different manifestations and complications of these diseases such as inefficiency of energy metabolism, vascular lesions, atherosclerosis, myocardial fibrosis, neuropathy, and nephropathy, among others. However, despite the advances in models and studied mechanisms, there is currently insufficient information on antioxidant treatments for these pathologies [16,24].

## 7. Role of SGLT2i

T2DM is a cardiometabolic disease that has been on the rise in recent times. Furthermore, it predisposes one to microvascular and macrovascular complications, as well as with respect to doubling the cardiovascular risk. Indeed, T2DM independently increases the risk of HF with respect to coronary artery disease and arterial hypertension; a fact that was correlated thanks to the Framingham study [25]. On the other hand, HF has become the new global pandemic of recent times, as it is a growing public health problem worldwide. To date, there has been no therapy that can be shown to reduce mortality and morbidity in patients with HF and T2DM [25].

Currently, four types of drugs are recommended for the purposes of treating HF with a reduced ejection fraction (HFrEF) in order to reduce the risk of hospitalization and death (with a level of evidence IA): angiotensin-converting enzyme (ACE) inhibitors; beta-blockers; mineralocorticoid antagonists; and SGLT2i. Indeed, sacubitril-valsartan, a neprilysin inhibitor, possesses an IB level of evidence that is recommended as a substitute for ACEIs in order to reduce the risk of hospitalization and death with respect to HF. It is believed that during the onset of HF, the consumption of free fatty acids increases, but later the use of this metabolite is affected as the extent of HF progresses; as such, other methods of utilizing energy appear. In addition, it has been observed that the expression of the GLUT4 transporter at the cardiomyocyte level also decreases [25].

Since SGLT2i emerged, several studies have revealed distinctive cardiovascular benefits, first seen in trials looking for better options to treat T2DM. The first study of SGLT2i, EMPA-REG OUTCOME was published in 2015 and studied the long-term effect of empagliflozin vs. placebo in a randomized, double-blinded trial in patients with T2DM. After 3.1 years, the study showed a reduction in the combined outcome of myocardial infarction and stroke by 14%, cardiovascular death by 38%, and hospitalizations by 35%. Exhibiting for the first time a glucose-lowering agent that could lower the risk of death from cardiovascular disease. In terms of the renal outcomes, there was a 39% reduction in the onset and progression of nephropathy in addition to delaying the albuminuria progression by 38% [1].

Similar reports were published in 2017 on the CANVAS program, which compared the effects of canagliflozin vs. placebo for 3.6 years in patients with T2DM and high cardiovascular risk. The primary outcome in this study was a composite of nonfatal stroke, death from cardiovascular causes, and nonfatal myocardial infarction. The rate of said outcome was lower with canagliflozin compared to placebo and even though the renal outcomes were not seen as statistically significant there was a delay in the progression of albuminuria. In addition, the results showed an attenuated decline in the estimated glomerular filtration rate eGFR, the need for renal-replacement therapy, and death from renal causes, suggesting a nephroprotective effect. There was also the report of a higher risk of amputation in the group that was treated with canagliflozin [26].

Subsequently, published in 2019 the DECLARE TIMI 58 trial evaluated dapagliflozin vs. placebo in patients with T2DM that had or were at risk for atherosclerotic cardiovascular disease in a period of 4.2 years. The primary safety outcome was a compilation of major adverse cardiovascular events (MACE), composed of cardiovascular death, myocardial infarction, or ischemic stroke. The primary efficacy outcomes were MACE and cardiovascular death or hospitalization due to HF. Secondary efficacy outcomes were renal parameters, such as a decrease in estimated glomerular filtration rate, new end-stage renal disease, and death from a renal or cardiovascular cause. Although it failed to show a lower rate of MACE, it did result in a lower rate of cardiovascular death or hospitalization for HF and was associated with a 24% reduction in cardiorenal outcome [2].

After large trials initially looking into safety outcomes with SGLT2i, which surprisingly resulted in cardiovascular benefits and mortality rate reduction, these drugs gained attention, mainly due to their unclear mechanism of action which seems independent of their hypoglycemic effect. These drugs evolved from being considered antidiabetic treatment to inspiring clinical trials for HF, both in T2DM and non-diabetic patients. Published in 2021, the EMPA-TROPISM clinical trial investigated the effects of empagliflozin, in non-diabetic HF for 6 months. The primary endpoint was to analyze changes in LV end-diastolic and systolic volume assessed by cardiac magnetic resonance. While the secondary endpoints consisted of a composite of changes in LV mass, ejection fraction, peak oxygen consumption in the cardiopulmonary exercise test, 6 minutes walk test, and quality of life. After meeting their primary and secondary endpoints, the trial concluded that empagliflozin administration to non-diabetic HFrEF significantly improves LV volumes, LV mass, LV systolic function, functional capacity, and quality of life when compared with placebo. This clinical trial observations strongly support a role for SGLT2i in the treatment of HFrEF patients independent of their glycemic status [27,28,29,30].

In 2020, the EMPEROR-REDUCED TRIAL was published, where the use of empagliflozin vs. placebo was assessed in patients with HFrEF, independently of their diabetic status. The primary event was cardiovascular death or hospitalization for worsening HF, which occurred in 19.4% of patients in the treatment group vs. 24.7% with placebo. Safety was highlighted in both groups as there were no complications such as ketoacidosis or hypoglycemia events [31].

Influence of sex on the effects of empagliflozin in HFpEF (Emperor preserved). Empagliflozin reduced the risk of CV death or hospitalization for HF to a similar degree in both sexes. Sex did not modify the relationship between empagliflozin and outcomes across ejection fraction groups. Improvement in Kansas City Cardiomyopathy Questionnaire Clinical Summary Score to a similar extent in both sexes. Similar effects on outcomes and health status in women and men with HFpEF [32].

SGLT2i have come a long way proving beneficial on CV and renal outcomes independently of diabetic status. The benefits go far beyond glycemic control, and both cardio and nephroprotection are underpinned by diverse mechanisms. From the activation of tubule glomerular feedback and the consequent reduction in hyperfiltration to the improvement of hypoxia and oxidative stress in the renal cortex [33].

SGLT2i have also been shown to inhibit hepcidin and limit podocyte damage. Likewise, they improve cardiac metabolism and bioenergetics and reduce necrosis and cardiac fibrosis, and the production of adipokines, cytokines, and epicardial adipose tissue mass. The analysis of subgroups of individuals with specific diseases such as IgA nephropathy has confirmed this solid effect on renal outcomes [33].

The benefits of SGLT2i were so evident that they were observed a few days after the onset of treatment and were maintained for more than three years, especially in patients with HF. Among the improvements observed, this drug managed to reduce cardiovascular (CV) mortality from all causes, including the number of hospitalizations by HF, as well as the progression of chronic kidney disease. Currently, however, its beneficial mechanisms are not fully understood; having said that, it is known that it acts upon hyperglycemia, the inflammatory response, blood pressure, visceral adiposity, injury, apoptosis, and cardiac remodeling, among other effects [31,34].

There are many hypotheses regarding their mechanism of action, one of them being that SGLT2i are drugs that are capable of increasing KB and FFA, which will thus serve as the necessary fuel, instead of glucose, for cardiac muscle requirements [35]. In an experimental study carried out on 14 non-diabetic pigs that were subjected to an ischemic myocardial injury, these pigs were randomly treated with empagliflozin vs. placebo for two months. Following this, an improvement in cardiac remodeling, left ventricular systolic function, and neurohumoral responses were evidenced in comparison to the control group. In addition, the empagliflozin-treated group exhibited changes in energy metabolism, which were caused by a decreased myocardial glucose uptake toward the use of KB, and FFA, as well as the branched-chain amino acids through increased gluconeogenesis. Indeed, this was noted to be similar to what occurs, via prolonged fasting, in ketogenesis [13,35].

The failing heart demands enormous amounts of energy and, for this reason, requires changes in energy metabolism [35]. Kim L Ho et al. evaluated cardiac metabolism in the hearts of mice that were subjected to HF through the transverse aortic constriction model in order to generate a pressure overload-induced cardiac hypertrophy [14]. The purpose was to analyze whether the increase in ketone bodies, induced by the perfusion of β-hydroxybutyrate (βOHB), improved cardiac efficiency by increasing its availability to generate energy through its oxidation without compromising the metabolism of the other energy pathways (i.e., ACGL and glucose). When these hearts are exposed to 600 μM βOHB, they can contribute more than 20% of the heart’s energy output. Thus, increasing ketones from 200 μM to 600 μM increases ketone oxidation in the failing heart, thereby contributing to greater overall energy output. Nonetheless, this is achieved without improving cardiac efficiency. In addition, the extra energy production obtained by increasing ketone oxidation does not come at the expense of glucose or fatty acid oxidation. In addition, interestingly, it also resulted in a significant upregulation with respect to the contribution of glucose oxidation to energy production [14].

One mechanism postulated to explain the benefits is that SGLT2i improves ventricular loading, secondary to a reduction in preload, primarily due to a diuretic and natriuretic effect. Inhibiting SGLT2i in the proximal tubule promotes glucosuria and natriuresis, which is a stimulus for tubuloglomerular feedback. This will lead to constriction of the afferent arteriole which will lower intraglomerular hypertension. Said diuretic effect seems to explain the benefits, however, other diuretics have not changed the prognosis in HF.

When comparing dapagliflozin to other diuretics, it was noted that SGLT2i reduced plasma volume, increased erythrocyte mass, and reduced sodium and interstitial fluid with little or no effect on blood volume. Demonstrating the ability SGLT2i have to selectively lower interstitial fluid. These drugs improve endothelial function and aortic stiffness, in addition to promoting vasodilation by activating voltage-gated potassium channels and protein kinase G. There is also SGLT2i’s property of being uricosuric [36].

A double-blind, randomized, placebo-controlled study assessed renal oxygenation as a possible mechanism for empagliflozin renal benefits in non-diabetics. Following 45 subjects for 1 month, urine sampling, renal ultrasound, and blood oxygenation level-dependent magnetic resonance imaging were performed both at baseline and after 1 month of initiating treatment. Even though there was no change in cortical and medullary renal oxygenation, it was found that empagliflozin reduced serum uric acid levels, and blood pressure decreased significantly. Given that hyperuricemia is a known risk factor for both CV and kidney disease, this could support a possible mechanism for their protective properties, and the fact that blood pressure may also be mediated by uric acid levels, which in turn may be regulated by ketogenesis [37].

There is increasing data suggesting SGLT2i improves cardiac energy metabolism and substrate efficiency by which cardiac output and efficiency improve. Under conditions such as T2DM and HF, the metabolic flexibility of the heart is impaired. SGLT2i increases the production of ketone body β-hydroxybutyrate (βOHB), switching the fuel to a less expensive myocardial source of energy. It has been suggested this happens through reducing ketone body levels being excreted by the kidneys and as a way to increase glucagon levels. βOHB “superfuel” is preferably oxidized in the heart than glucose and fatty acids, improving cardiac function and mechanical efficiency [36].

Animal studies showed empagliflozin increased myocardial ketone consumption and lowered lactate production and glucose consumption of the heart. While others hypothesize βOHB increased production inhibits histone deacetylase and prevents prohyperthropic transcription pathways. It could also be that lowering βOHB oxidation leads to a reduction in the acetyl-CoA that comes from ketone oxidation, hence promoting the oxidation of glucose-derived pyruvate [36,37].

An underrated mechanism of the cardiorenal benefits is that SLGT2i attenuates the sympathetic nervous system activity in diabetic patients. This proposed theory is based on the observation that this drug lowers BP without raising HR, suggesting a reduction in sympathetic overactivity [38].

When the kidney is damaged, the afferent renal nerve sends a signal to the brain increasing sympathetic outflow, as a compensatory mechanism. If it stays elevated for a long period of time, it could lead to attenuated blood flow to the kidneys and atherosclerosis, resulting in decreased renal function. SGLT2i provides CV protective effects by reducing renal afferent nerve activity, which in turn inactivates SNS outflow that will prevent generalized sympathetic activation. It has been seen there is an improvement in cardiac nerve activity without the side effects [38].

Another proposed mechanism is that the “fasting-mimicry” condition induced by the glycosuria effect of the SGLT2i leads to a negative energy balance. This in turn would explain the cardio-protective effects by activating the SIRT1/AMPK signaling pathway, which ends in the suppression of Akt/mTOR. This induces a metabolic state, similar to prolonged fasting, which involves several benefits: reduction of inflammation and oxidative stress, stabilization of mitochondrial function, and increase in both autophagy and contractile activity [39].

An important pathway to mention is the novel inflammation /NHE/[Na^+^]_c_/ROS pathway. Uthman et, al. studied how can SGLT2i can reduce inflammation, reverting oxidative stress. The hypothesis was that if TNF-α activates the Na^+^/H^+^ exchanger (NHE) and raises cytoplasmic Na^+^ ([Na^+^]_c_), this will lead to reactive oxygen species (ROS) production, then empagliflozin could reduce inflammation by lowering [Na^+^]_c_ and ROS. After incubating human umbilical vein endothelial cells (HUVECs) and human coronary artery endothelial cells (HCAECs) with 10 ng/mL TNF-α, 1 µM Empagliflozin or the NHE inhibitor cariporide (CARI, 10 µM); they corroborated their hypothesis. Empagliflozin reduced TNF α-induced ROS in both cells by inhibiting NHE and lowering [Na^+^]_c_ levels [40].

Sexual differences play an important role in the metabolic and molecular mechanisms underlying CV and kidney disease. Affecting their onset and progression, especially in T2DM and diabetic kidney disease (DKD). Women are at a higher CV risk conferred by the metabolic syndrome [41], in addition, they have a higher prevalence of ESKD and concomitant risk factors such as hypertension accelerates end-organ damage to a greater degree compared to men [42]. While post-menopausal women are the ones at higher risk for DKD development, men with either T1DM or T2DM are at a greater risk of DKD than pre-menopausal women. Even with these gender-related differences, women are still under-represented in clinical trials [41,42].

Research on rats found there are sex differences in the SGLT2 transcripts, white females having the higher expression. Further suggesting that an increment in the reabsorption of water, sodium and glucose increases parallel to the number of transporters. In regards to the compelling treatment, SGLT2i similar reports have been observed in both sexes [42].

## 8. Role of SGLT2i at the Renal Level—Chronic Kidney Disease

The glomerular damage caused by progressive proteinuria continues to be a challenge in the management of this pathology, which is why the objective has been to develop therapies that reduce proteinuria in order to protect the epithelial cells at the renal proximal tubule level. In regard to chronic kidney disease (CKD) patients, SGLT2i have been observed to offer renal protection, especially in subjects with DKD. However, having said this, the mechanism by which it does so is not entirely clear. Furthermore, recent evidence from large clinical trials has shown that SGLT2i reduces the risk of HF and improves renal outcomes in people with T2DM, in addition to delivering its hypoglycemic effect [2].

Issei Tomita et al. confirmed, via a study in knockout mice, that if non-proteinuric CKD submits to a high-fat diet for the purposes of ApoE, then SGLT2i offers renoprotection, as mediated by an increase in KB [43]. In that same study, it was observed that ATP production changed from lipolysis in order to be dependent on ketolysis, which was due to the hyperactivation of the rapamycin-responsive complex 1 (mTORC1) in the affected kidneys. Furthermore, empagliflozin was found to increase endogenous KB levels, and the use of 1,3-butanediol, which is a KB precursor, prevented renal ATP breakdown and organ damage in mice [43].

The protective effect of empagliflozin was eliminated by the deletion of the *Hmgcs2* gene, which encodes a protein that belongs to the HMG-CoA synthase family—a mitochondrial enzyme that catalyzes the first reaction of ketogenesis. In addition, KB suppresses proteinuria and the podocyte damage that is related to the mTORC1 expression in diabetic rats. Therefore, the results show that the renal protection associated with SGLT2i is mediated by an increase in KB (which inhibits and regulates mTORC1 hyperactivation), thereby leading to impaired renal lipolysis, as well as its consequent renal damage, which occurs in proteinuric and non-proteinuric DKD [43].

DAPA-CKD was published in 2020, after being discontinued early with a median follow-up of 2.4 years, due to the efficacy observed with dapagliflozin in patients with eGFR of 25 to 75 mL per minute per 1.73 m^2^ of body surface area and a urinary albumin-to-creatinine ratio of 200 to 5000. The primary outcome was a combination of a sustained decrease in eGFR of at least 50%, end-stage KD, and death from renal or CV causes. The primary outcome occurred significantly lower in the treatment group with 9.2%, compared to 14.5% in the placebo group. The effects were similarly observed in patients with and without T2DM, data that supports their mechanism being independent of glycemic levels [44].

The latest KDIGO guidelines of 2022 recommend that most patients with T2DM and CKD who have an eGFR ≥ 20 mL/min per 1.73 m^2^ should initiate an iSGLT2 as part of their therapeutic regimen. Many patients with T2DM require more than one antidiabetic agent to achieve glycemic targets. The combination of these drugs is convenient because their mechanism of action differs, therefore there is no risk of hypoglycemia. Despite the fact that glycemic targets are achieved with metformin, it is recommended to add an SGLT2 to reduce the progression of CKD and CV disease [45,46,47,48,49].

## 9. Hemodynamic Mechanisms of SGLT2 Inhibitors

Several clinical studies have shown a significant decrease in hospitalization with respect to HF after the use of SGLT2i, which suggests a relationship that is directly proportional to the improvement in hemodynamic status [50,51,52].

We know that SGLT2i reduces blood pressure (BP) by around 4.0/1.6 mmHg without generating a reflex increase in the heart rate. This, therefore, suggests that the sympathetic nervous system (SNS) is not activated and could even be inhibited. Indeed, the activation of the SNS is deleterious with respect to HF. Further, it is frequently accompanied by poor clinical outcomes. In a global context, when compared to other agents that can induce SNS activation, SGLT2i should be more beneficial for the treatment of HF patients [53,54].

On the other hand, if we compare the gradual lowering of glucose with the use of SGLT2i in patients with CKD, the effect with respect to lowering BP is similar in patients with different degrees of renal involvement, even in those with a greatly impaired glomerular filtration rate (eGFR), so much so that several theories have been postulated in this regard. Evidence has suggested that the antihypertensive effect could be secondary to a mechanism of volume reduction due to diuresis and natriuresis. Additionally, there are other effects, such as the loss of calories, as well as the decrease in fat mass and weight loss. However, these are secondary to the increase in diuresis and glycosuria, which would also contribute to the decrease in BP [55].

Recent studies suggest that the ketogenic properties of SGLT2i, even when independent of renal function, could also explain their antihypertensive effects. However, the decrease in BP observed with SGLT2i does not maintain a proportional relationship with the rapid, effective, and strong improvement found in the clinical results, which is, comparatively, of little relevance when compared to the results found with antihypertensive drugs [12,56].

Under physiological conditions, the glomerular filtration of Na^+^ and glucose takes place in the S1 and S2 segment of the proximal convoluted tubule. By inhibiting the reabsorption of the previously mentioned ions, SGLT2i promotes natriuresis and reduces the extracellular fluid, including plasmatic volume. Therefore, SGLT2i produces an early increase in urine volume during the first few days after beginning treatment, after which the urine volume gradually returns to a baseline level over several weeks, and, finally, a 7.3% reduction in plasma volume is observed after 12 weeks [57].

Unlike loop diuretics, SGLT2i tends to remove fluid from the interstitial space, inducing little effect on the vascular space and thus resulting in an increased electrolyte-free water clearance. Considering the fact that—in HF—there is an increased circulating volume load, with inadequate arterial perfusion due to impaired cardiac function, interstitial volume reduction may be more favorable for HF patients. For that reason, it is likely that natriuresis and a reduced volume load will explain, in part, the protective effects of SGLT2i in HF [5].

At the blood vessel level, arterial stiffness, which is associated with morbidity and mortality, is an important predictor of HF due to the fact that it can lead to increased cardiac output and a further deterioration of cardiac function. Certain cardioprotective agents, such as RAAS inhibitors, decrease arterial stiffness, and cardiac output, and improve CV outcomes. In addition, endothelial function also plays an important role in the maintenance of myocardial function, hemodynamics, systemic circulation, and pulmonary circulation. When there is a dysfunction at the endothelial level, there are alterations in nitric oxide (NO) production and utilization, thus further increasing vascular resistance [57,58].

Dapagliflozin was able to significantly reduce aortic pulse wave velocity (PWV), which is the gold standard parameter for arterial stiffness. Similarly, in subsequent randomized controlled studies, with empagliflozin and canagliflozin being compared with placebos, a significant decrease in PWV was observed. These results suggested that SGLT2i may alleviate arterial stiffness, in addition to improving endothelial function. These beneficial effects may be mediated by an increased NO production, reduced oxidative stress, as well as the activation of voltage-gated potassium (K^+)^ channels and protein kinase G. However, unlike the three, well-recognized SGLT2i (i.e., empagliflozin, dapagliflozin, and canagliflozin), luseogliflozin did not show a similar beneficial effect with respect to arterial stiffness. Indeed, further assessment is required in order to determine if this may indicate that the improvement in arterial stiffness with SGLT2i is due to a specific drug, rather than due to a class effect, or due to the fact that the sample size and duration of the luseogliflozin study were limited [58].

## 10. Conclusions

Even though at first, the mechanisms of SGLT2i were believed to be ligated to glucose levels, over the years, it has been demonstrated that their mechanism of action is beyond simply that function alone. In addition, with several hypotheses on the rise and provided the fact that their benefits are observed independently of hyperglycemia, they are a very promising therapy option with respect to treating vascular disease for the diabetic and non-diabetic populations.

The use of SGLT2i at the renal level has also shown that they generate a beneficial metabolic alteration, which is obtained by reducing the glucotoxicity that is induced by glycosuria. Together with the reduction in perivisceral fat in various organs, this then leads to the use of free fatty acids in the initial stages of the affected heart. As such, this will cause an increase in the production of ketoacids, which are a more available energy fuel at the cellular level.

During HF, glucose consumption is increased in comparison to FFA, that is to say, the proceeding metabolic pathway changes to anaerobic glycolysis, thereby producing a small reduction in ATP. Despite this, the cellular demand for ATP remains constant, thus resulting in an energy deficit. Therefore, this alternate route cannot sustain the already existing energy demands.

However, the strong hemodynamic, anti-inflammatory, myocardial remodeling, and metabolic benefits of SGLT2i cannot fully explain the rapid improvement and efficacy of clinical outcomes that precede the decrease in morbidity and mortality in patients with HF. These mechanisms improve the prognosis, hope, and quality of life of these patients, regardless of the left ventricular ejection fraction. Although it is an alternate pathway that increases available energy, but not cardiac efficiency, future research will clarify other mechanistic options that explain the different substrates within the global context of understanding cardiac metabolic flexibility and ketogenesis. In order to better understand the effects, and thus, be able to use the appropriate therapy, targeting the different mechanisms involved in the most predominant CV and renal pathologies is required.

Additionally, even though the mechanism of SGLT2i is not fully understood, their vast benefits make it of incredible importance to continue to utilize them, as well as research them further.
